# First Documented Ivor Lewis Oesophagectomy for Oesophageal Squamous Cell Carcinoma in Somalia: A Case Report From a Resource‐Limited Setting

**DOI:** 10.1155/cris/7934976

**Published:** 2026-07-08

**Authors:** Resul Nusretoğlu, Elmi Kasim Osman

**Affiliations:** ^1^ Department of General Surgery, Mogadishu Somali-Turkish Training and Research Hospital, Mogadishu, Somalia

**Keywords:** curative-intent surgery, global surgery, Ivor Lewis oesophagectomy, oesophageal squamous cell carcinoma, R1 resection, resource-limited setting, Somalia

## Abstract

**Background:**

Oesophageal squamous cell carcinoma is associated with poor prognosis, particularly in resource‐limited settings where access to advanced staging, multi‐modal oncology treatment and complex surgical care is constrained. Ivor Lewis oesophagectomy is an established surgical approach for selected mid‐ and distal oesophageal tumours, but its implementation requires specialised surgical, anaesthetic and peri‐operative support. To the best of our knowledge, this is the first documented report of Ivor Lewis oesophagectomy for oesophageal squamous cell carcinoma performed in Somalia.

**Case Presentation:**

A 42‐year‐old female presented with a 3‐month history of progressive dysphagia affecting both solid and liquid foods. Upper gastrointestinal endoscopy demonstrated a mid‐oesophageal mass, and biopsy confirmed Grade 1 well‐differentiated squamous cell carcinoma. Contrast‐enhanced CT of the chest and abdomen showed a mid/subhilar oesophageal tumour with luminal narrowing and proximal oesophageal dilatation, without radiological evidence of distant metastasis. Because PET‐CT and endoscopic ultrasound were unavailable, precise T and N staging could not be determined; therefore, the disease was clinically staged as cT2–T3 Nx M0 and considered resectable. Neoadjuvant chemoradiotherapy was considered, but timely access to standardised treatment was limited. Upfront curative‐intent surgery was therefore selected after assessment of resectability, patient fitness and available local resources. The patient underwent open Ivor Lewis oesophagectomy with gastric conduit formation, limited lymphadenectomy, feeding jejunostomy placement and hand‐sewn intra‐thoracic oesophagogastric anastomosis. Operative time was 5 h, estimated blood loss was 800 mL and there were no intra‐operative complications.

**Discussion:**

Post‐operative CT demonstrated small bilateral pleural effusions, mild pneumothorax and pneumomediastinum, which were managed conservatively without reoperation or invasive intervention. A post‐operative day‐7 contrast swallow study showed no anastomotic leak or obstruction. The final histopathology confirmed Grade 1 well‐differentiated squamous cell carcinoma with an R1 resection margin, indicating microscopic tumour involvement at the margin. The exact lymph node yield and number of metastatic lymph nodes were not clearly documented, limiting complete pathological nodal staging and assessment of lymphadenectomy adequacy. Intra‐operative frozen‐section margin assessment was not performed because this facility was not reliably available. At 7 weeks’ follow‐up, the patient was tolerating oral intake with no clinical evidence of anastomotic leak or stricture, and oncology review was arranged for consideration of adjuvant treatment and structured surveillance.

**Conclusion:**

This case demonstrates the technical feasibility of open Ivor Lewis oesophagectomy for oesophageal squamous cell carcinoma in a resource‐limited setting. However, the R1 margin, incomplete nodal assessment, limited staging modalities and short follow‐up mean that this case should be interpreted as a technically successful, curative‐intent procedure rather than evidence of complete oncological clearance. Long‐term oncological follow‐up remains ongoing.

## 1. Introduction

Oesophageal carcinoma remains one of the most aggressive gastrointestinal malignancies worldwide, with high mortality rates largely attributable to late presentation and limited access to curative treatment in low‐ and middle‐income countries (LMICs) [1, 2]. Surgical resection, as part of a multi‐modal treatment strategy, offers the best chance of long‐term survival for patients with resectable disease; however, access to oesophagectomy remains highly inequitable across regions, particularly in fragile and resource‐limited health systems [3].

The Ivor Lewis esophagectomy is a well‐established operative approach for mid‐ and distal oesophageal carcinoma, combining abdominal and thoracic phases with an intra‐thoracic anastomosis. In high‐income settings, this procedure is routinely performed in specialised centres supported by advanced anaesthesia, intensive care and multi‐disciplinary oncology services [4, 5]. In contrast, the technical complexity of the operation, coupled with the need for robust peri‐operative support, has limited its adoption in low‐resource environments, where concerns regarding post‐operative morbidity and mortality often preclude curative surgical intent [6].

In Somalia, prolonged conflict, shortages of trained surgical personnel and limited critical care capacity have historically restricted access to complex oncological surgery. As a result, patients with oesophageal carcinoma have largely been managed with palliative measures or referred abroad when it is financially and logistically feasible. To our knowledge, no published reports have documented the performance of Ivor Lewis esophagectomy within Somalia, reflecting both the systemic challenges and the absence of locally available definitive surgical services for this disease [7].

The establishment of oesophageal resection services in such settings represents an important step toward strengthening surgical capacity and expanding access to essential cancer care [8]. We, therefore, report the first documented case of Ivor Lewis esophagectomy for oesophageal carcinoma performed in Somalia. This case report aims to demonstrate the feasibility and early safety of the procedure, describe the peri‐operative challenges encountered and share practical insights relevant to surgeons working in similar low‐resource contexts.

## 2. Case Presentation

A 42‐year‐old female patient presented with a 3‐month history of progressive dysphagia affecting both solid and liquid foods. Following clinical assessment, she underwent upper gastrointestinal endoscopy, which demonstrated a mid‐oesophageal mass (Figure 1). Endoscopic biopsy confirmed Grade 1 well‐differentiated squamous cell carcinoma of the oesophagus.

**Figure 1:**
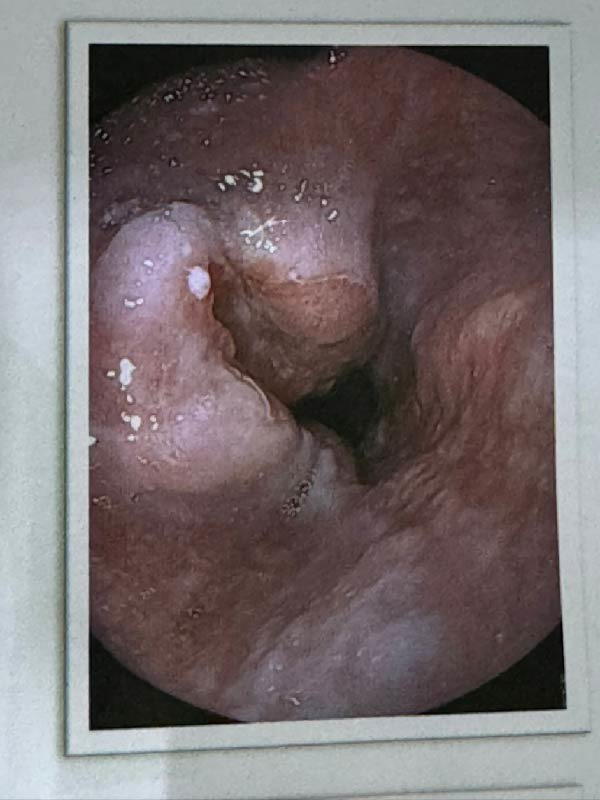
Upper gastrointestinal endoscopy showing an oesophageal mass.

Pre‐operative staging was performed using contrast‐enhanced CT of the chest and abdomen. CT demonstrated short‐segment circumferential wall thickening of the mid/subhilar oesophagus with associated luminal narrowing and proximal oesophageal dilatation, consistent with oesophageal malignancy (Figure 2A). There was no radiological evidence of distant metastatic disease. Because endoscopic ultrasound and PET‐CT were not available, precise clinical T and N staging could not be determined. Based on the available imaging, the disease was clinically staged as cT2–T3 Nx M0 and was considered resectable.

**Figure 2:**
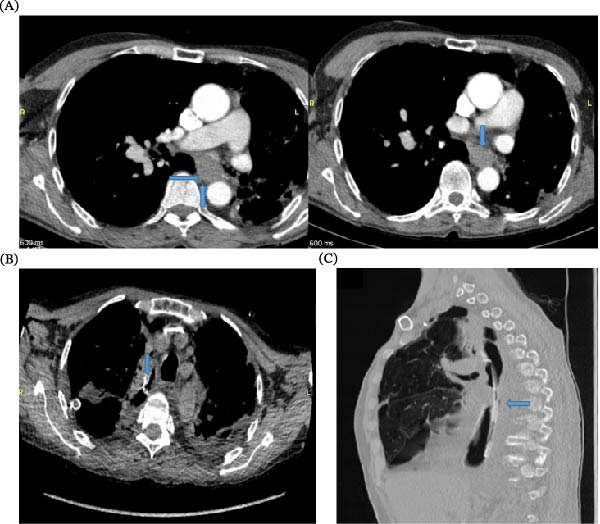
(A) Preoperative contrast‐enhanced CT showing short‐segment circumferential wall thickening of the mid/subhilar oesophagus with luminal narrowing and proximal oesophageal dilatation, suspicious for oesophageal malignancy. (B) Demonstrates mild oesophageal dilatation with visible suture lines in the mid‐oesophagus, consistent with postoperative changes following an Ivor Lewis procedure. (C) The sagittal image shows mild oesophageal dilatation with a nasogastric tube in situ. Additional postoperative findings include bilateral pleural effusions, mild pneumothorax, and pneumomediastinum.

The case was reviewed by the available surgical and oncology teams. Neoadjuvant chemoradiotherapy was considered, as multi‐modal therapy is commonly recommended for resectable locally advanced oesophageal cancer. However, because of limited local access to timely chemoradiotherapy and the practical constraints of delivering standard neoadjuvant treatment in this resource‐limited setting, upfront surgical resection was selected as the most feasible curative‐intent option. This decision was made after assessment of tumour resectability, the patient’s physiological fitness for major surgery and informed discussion with the patient.

The patient subsequently underwent open Ivor Lewis oesophagectomy. The abdominal phase involved mobilisation of the stomach and creation of a gastric conduit along the greater curvature using linear staplers (Figure 3). A limited lymphadenectomy was performed, including abdominal and mediastinal nodal sampling. The exact total lymph node yield and number of metastatic lymph nodes were not clearly documented in the available final pathology record, and therefore, a complete pathological N stage could not be assigned. Pyloric drainage was not performed. A feeding jejunostomy was placed to support post‐operative nutrition.

**Figure 3:**
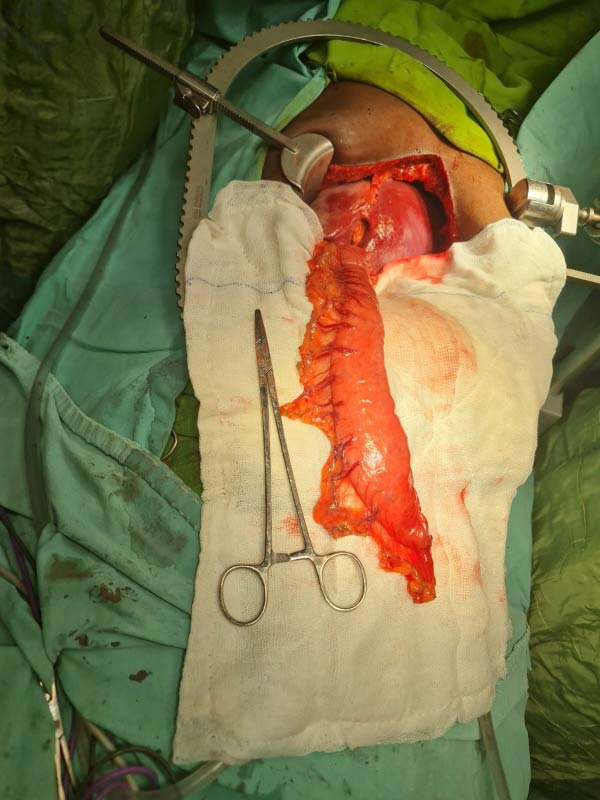
Intra‐operative image showing creation of the gastric conduit along the greater curvature using linear staplers.

The thoracic phase was completed through a right thoracotomy. The tumour‐bearing oesophageal segment was resected, followed by intra‐thoracic oesophagogastric anastomosis using a hand‐sewn technique (Figure 4). Two drains were placed adjacent to the anastomotic site. The total operative time was 5 h, and the estimated blood loss was 800 mL. There were no intra‐operative complications.

**Figure 4:**
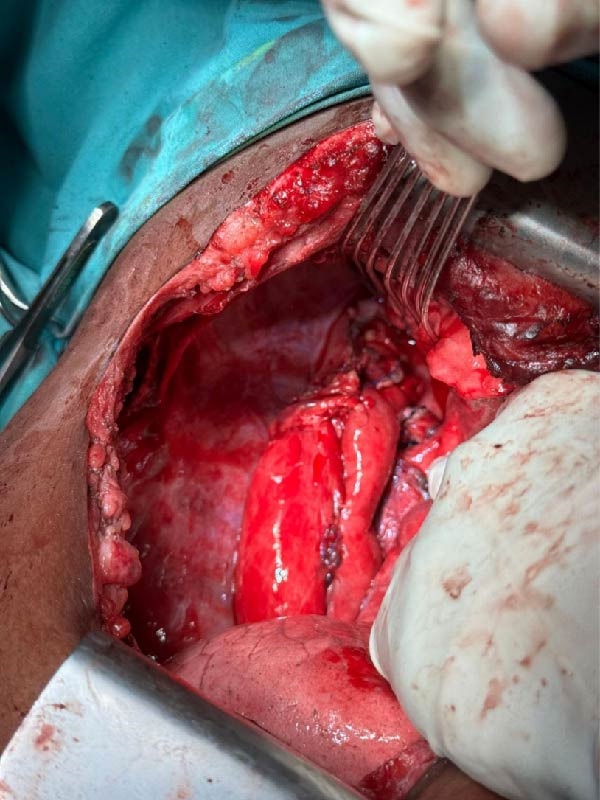
Intra‐operative image showing right thoracotomy and completion of intra‐thoracic oesophagogastric anastomosis.

Post‐operatively, the patient was monitored closely. Post‐operative CT demonstrated early post‐operative changes, including small bilateral pleural effusions, mild pneumothorax and pneumomediastinum (Figure 2B,C). These findings were managed conservatively and did not require re‐operation, chest tube reinsertion, or other invasive interventions. The figure file describes Figure 2B,C as post‐operative images showing visible suture lines, nasogastric tube in situ, pleural effusions, pneumothorax and pneumomediastinum.

On post‐operative day 7, a contrast swallow study using barium showed no evidence of anastomotic leak or obstruction (Figure 5). The patient subsequently tolerated oral intake and was discharged in stable condition, with outpatient surgical and oncology follow‐up arranged. Figure 5 in the submitted figures is labelled as the post‐operative contrast swallow study, showing no leak or obstruction.

**Figure 5:**
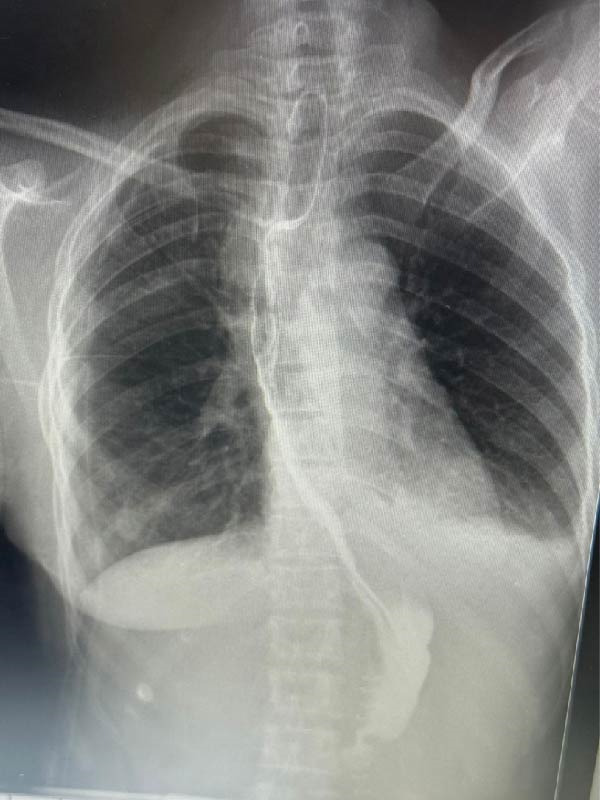
Post‐operative contrast swallow study showing no evidence of anastomotic leak or obstruction.

Final post‐operative histopathology confirmed Grade 1 well‐differentiated squamous cell carcinoma of the oesophagus with an R1 resection margin, indicating microscopic tumour involvement at the margin. No distant metastasis was identified. However, the final pathology report did not provide a complete pathological nodal stage, and the exact lymph node yield and number of metastatic lymph nodes could not be fully verified from the available records. Intra‐operative frozen‐section assessment of the resection margins was not performed because this facility was not reliably available at the time of surgery. Macroscopically, the resection margins were considered grossly clear intra‐operatively, but microscopic margin involvement was subsequently identified on the final histopathological examination. In view of the R1 margin status and incomplete nodal staging, the patient was referred for post‐operative oncology review to consider adjuvant treatment and structured surveillance.

At 7 weeks’ follow‐up, the patient was tolerating oral intake and had no clinical evidence of an anastomotic leak or stricture. She remained under surgical follow‐up and was referred for post‐operative oncology review in view of the R1 margin status. Long‐term oncological follow‐up remains ongoing.

## 3. Discussion

This case report represents the first documented experience of Ivor Lewis esophagectomy for oesophageal carcinoma performed in Somalia and demonstrates the feasibility of undertaking complex oncological surgery in a resource‐limited setting [3]. The patient underwent successful curative resection with satisfactory early post‐operative outcomes despite limitations in infrastructure, critical care capacity and access to advanced peri‐operative technologies [2].

Current management of resectable locally advanced oesophageal squamous cell carcinoma commonly involves multi‐modal treatment, including neoadjuvant chemoradiotherapy where available. The CROSS trial demonstrated that pre‐operative chemoradiotherapy followed by surgery improved survival compared with surgery alone in patients with potentially curable oesophageal or oesophagogastric‐junction cancer [9].

In this case, neoadjuvant treatment was considered; however, timely access to standardised chemoradiotherapy was limited. Upfront surgery was therefore selected as the most feasible curative‐intent option after the assessment of resectability, patient fitness and available local resources. This reflects the pragmatic decision‐making required in resource‐limited settings, where delays or lack of access to neoadjuvant therapy may prevent patients from receiving definitive treatment.

Surgical radicality, lymph node yield and margin status are central considerations when assessing the oncological adequacy of oesophagectomy. In standard oesophageal cancer surgery, the aim is not only the removal of the primary tumour but also the achievement of an R0 resection with adequate locoregional lymphadenectomy for staging and disease control. Lymph node yield is an important quality indicator because it improves the accuracy of pathological nodal staging, reduces the risk of understaging and provides prognostic information. Several guidelines and surgical oncology reviews recommend that at least 15–16 regional lymph nodes should ideally be examined following curative‐intent oesophagectomy, although the achievable yield may vary according to tumour location, operative approach, use of neoadjuvant therapy and local pathology resources [10].

In the present case, only a limited lymphadenectomy with abdominal and mediastinal nodal sampling was performed. This reflected the early stage of local programme development, the absence of advanced staging modalities such as PET‐CT and endoscopic ultrasound, and the need to balance oncological radicality against operative safety in a resource‐limited setting. The final pathology report did not provide a complete pathological nodal stage, and the exact total lymph node yield and number of metastatic lymph nodes could not be fully verified from the available records. This represents an important limitation of the case, as the true nodal status and lymph node ratio could not be reliably assessed. Therefore, while the procedure demonstrated technical feasibility, the oncological adequacy of lymphadenectomy cannot be considered equivalent to that expected in high‐volume oesophageal cancer centres with standardised pathological reporting.

The final histopathology demonstrated an R1 resection margin, indicating a microscopic residual tumour. This finding is clinically significant because margin‐positive resection after oesophagectomy is associated with a higher risk of local recurrence and poorer survival compared with R0 resection [11].

Intra‐operative frozen‐section assessment of resection margins was not performed in this case because frozen‐section pathology was not reliably available at the time of surgery. The macroscopic resection margins were assessed intra‐operatively by the operating team and were considered grossly clear; however, microscopic margin involvement was only identified on the final histopathological examination. This limitation highlights an important challenge in resource‐limited surgical oncology, where the absence of real‐time pathological margin assessment may reduce the ability to modify the resection intra‐operatively.

In view of the R1 margin status and incomplete nodal staging, this operation should, therefore, be interpreted as a technically successful, curative‐intent oesophagectomy rather than definitive evidence of complete oncological clearance. The patient was referred for post‐operative oncology review to consider adjuvant treatment and structured surveillance. These findings also emphasise the need for future development of standardised oesophageal cancer pathways in Somalia, including access to PET‐CT or endoscopic ultrasound, multi‐disciplinary tumour board review, frozen‐section pathology where feasible, systematic lymphadenectomy, and structured pathology reporting of lymph node yield, metastatic nodes and margin status.

Oesophagectomy is recognised as a technically demanding procedure associated with significant morbidity and mortality, even in high‐volume centres with established multi‐disciplinary support [4]. Pulmonary complications and anastomotic leaks remain the most common causes of post‐operative morbidity and mortality following esophagectomy worldwide [5]. These risks are further amplified in low‐resource environments due to constraints in anaesthesia services, post‐operative monitoring and specialist nursing care [8].

Despite these challenges, the patient did not experience major early post‐operative complications, suggesting that acceptable outcomes are achievable with careful adaptation of surgical and peri‐operative practices [12]. Rigorous patient selection, focusing on resectable disease and adequate physiological reserve, was a key factor contributing to the success of this case [13]. Meticulous adherence to fundamental surgical principles, including careful dissection and construction of a tension‐free intra‐thoracic anastomosis, further reduced the risk of complications [4].

Close post‐operative observation and early recognition of potential complications played a critical role in compensating for the absence of advanced intensive care facilities [12]. This experience supports emerging evidence from other LMICs demonstrating that complex cancer surgery can be delivered safely when care pathways are adapted to local realities rather than directly replicating high‐income country models [8].

Beyond the individual patient outcome, the successful performance of this procedure has broader implications for surgical system strengthening in Somalia. Establishing local capacity for oesophageal cancer surgery reduces reliance on overseas referral, which is often financially prohibitive and inaccessible to most patients [7]. Furthermore, the introduction of complex oncological procedures can act as a catalyst for improvements in anaesthesia, peri‐operative nursing and post‐operative care, with potential benefits extending across other surgical specialties [14].

## 4. Limitations

This report has several limitations. First, it describes a single case and therefore cannot establish generalisable safety or oncological efficacy. Second, pre‐operative staging was limited to contrast‐enhanced CT because PET‐CT and endoscopic ultrasound were not available; therefore, precise clinical T and N staging could not be determined. Third, the final histopathology demonstrated an R1 resection margin, indicating microscopic residual disease. As a result, the procedure should be interpreted as technically successful and curative‐intent rather than oncologically complete.

Fourth, follow‐up remains short, and late complications, recurrence, disease‐free survival and overall survival cannot yet be assessed. Fifth, the exact lymph node yield and number of metastatic lymph nodes were not available from the final pathology record, limiting assessment of the pathological nodal stage, lymph node ratio and the oncological adequacy of lymphadenectomy. Finally, intra‐operative frozen‐section assessment of the resection margins was not performed because this facility was not reliably available, and the R1 margin was only identified on final histopathology.

## Funding

No funding was received for this manuscript.

## Ethics Statement

Ethical approval for the publication of this case report was granted by the Institutional Review Board of the Mogadishu Somali Turkish Training and Research Hospital.

## Consent

Written informed consent was obtained from the patient for participation and for the publication of the case details and any accompanying images.

## Conflicts of Interest

The authors declare no conflicts of interest.

## Data Availability

The data supporting the findings of this case report are included within the article. Additional clinical information is available from the corresponding author upon reasonable request, subject to patient confidentiality and ethical considerations.
